# Second-hand smoke exposure in adolescents in Latin America and the Caribbean: a pooled analysis

**DOI:** 10.1016/j.lana.2023.100478

**Published:** 2023-03-20

**Authors:** Antonio Bernabe-Ortiz, Rodrigo M. Carrillo-Larco

**Affiliations:** a CRONICAS Center of Excellence in Chronic Diseases, Universidad Peruana Cayetano Heredia, Lima, Peru; b Universidad Científica del Sur, Lima, Peru; c Department of Epidemiology and Biostatistics, School of Public Health, Imperial College London, London, UK; d Universidad Continental, Lima, Peru

**Keywords:** Second-hand smoking, Tobacco smoke pollution, Smoke-free environment, Adolescents

## Abstract

**Background:**

Second-hand smoke exposure is prevalent amongst adolescents, despite of being a preventable risk factor associated with unfavourable outcomes. The distribution of this risk factor varies by underlying determinants and public health officers need contemporary evidence to update policies. Using the most recent data available from adolescents in Latin America and the Caribbean (LAC), we described the prevalence of second-hand smoking.

**Methods:**

Pooled analysis of Global School-based Student Health (GSHS) surveys conducted from 2010 to 2018 was conducted. Two indicators were analysed based on information from the 7 days prior to the survey: a) any exposure to second-hand smoking (0 vs ≥1 days of exposure); and b) daily exposure (<7 vs 7 days). Prevalence estimates were carried out accounting for the complex survey design, and reported overall, by country, by sex, and by subregion.

**Findings:**

GSHS surveys were administered in 18 countries, yielding a total of 95,805 subjects. Pooled age-standardised prevalence of second-hand smoking was 60.9% (95% CI: 59.9%–62.0%) with no substantial differences between boys and girls. The age-standardised prevalence of any second-hand smoking varied from 40.2% in Anguilla to 68.2% in Jamaica, and the highest prevalence was in the Southern Latin America subregion (65.9%). Pooled age-standardised prevalence of daily second-hand smoking was 15.1% (95% CI: 14.2%–16.1%), and was higher in girls than boys (16.5% vs 13.7%; p < 0.001). The age-standardised prevalence of daily second-hand smoking ranged between 4.8% in Peru to 28.7% in Jamaica, and the highest age-standardised prevalence was in Southern Latin America (19.7%).

**Interpretation:**

The prevalence of any second-hand smoking is high among adolescents in LAC, though estimates changed substantially by country. While policies and interventions to reduce/stop smoking are implemented, attention should also be paid to avoid second-hand smoke exposure.

**Funding:**

Wellcome Trust International Training Fellowship (214185/Z/18/Z).

**Research in context**

**Evidence before this study**

Current worldwide evidence based on a literature search in PubMed (August 1st, 2022) with the following search strategy: “second-hand smoking” AND “survey” and “adolescents”, signals that, according to pooled data of the Global Youth Tobacco Survey, 62.9% of adolescents, aged between 12 and 16 years, has had any exposure to second-hand smoking during last seven days, whereas 32.5% reported a daily exposure. Second-hand smoking has been steadily growing in the Latin America and the Caribbean region. As a result, a more detailed analysis is needed in our region to appropriately implement and improve existing strategies.

**Added value of this study**

Our study expands existing literature in the region by updating prevalence results of second-hand smoking by country and by sex, but also among never smoker adolescents. Despite of the results variability and the implementation of the Framework Convention on Tobacco Control in several countries of the region, the prevalence of second-hand smoke exposure is high among adolescents, especially among females and never smokers.

**Implications of all the available evidence**

Our results highlight the need to strengthen policies to tackle the problem of second-hand smoking. Future research in the region should focus on defining the origin of such exposure (home, school, elsewhere) to implement appropriate strategies.

## Introduction

Whilst the estimated absolute number of subjects who died of second-hand smoke exposure decreased between 1990 and 2006, this number has gradually increased after that.^1^ Moreover, second-hand smoke exposure was the cause of about 603,000 premature deaths in 2004, and out of all deaths attributable to second-hand smoking, 28% occur in children and adolescents.^2^ In addition, second-hand smoking has slowly but steadily grown between 1990 and 2016 in Latin America and the Caribbean (LAC) region.^1^^,^^3^

Based on the most recent Global Youth Tobacco Surveys (GYTS), and with information of 142 countries, it was reported that 62.9% of adolescents, aged between 12 and 16 years, has had any exposure to second-hand smoking during last seven days, whereas 32.5% reported a daily exposure.^4^ In the same study and using data from 29 LAC countries, second-hand smoking and daily second-hand smoking were present in 53.6% and 22.5% of adolescents, respectively. Although the prevalence of second-hand smoking was considered high among adolescents, no difference between sexes was reported.^5^ Moreover, second-hand smoking may have a greater impact on never smokers, a group that has not been evaluated since 2008.^6^ In addition, second-hand smoking has been associated with high probability of being smoker, susceptibility to smoking, smoking initiation, and greater nicotine dependence.^7^^,^^8^ As a result, the information herein provided may give current local information useful for appropriate interventions.

Second hand smoking is a relevant modifiable (and preventable) risk factor,^9^ and it has been associated with increased risk of tobacco use, especially among young adolescents,^10^ with the subsequent impact on health outcomes.^11^, ^12^, ^13^, ^14^ As a result, the Framework Convention on Tobacco Control (FCTC) was developed by the World Health Organization (WHO) to reduce the increase of tobacco consumption, the escalation in smoking by children and adolescents, and the impact of all forms of advertising, promotion and sponsorship aimed at encouraging tobacco use.^15^^,^^16^ Many countries have signed this framework, but only one (Dominican Republic) has not done so, and some other (Argentina, Cuba and Haiti) did not ratify it. Moreover, strategies implemented by countries to tackle this public health concern are also variable,^17^ making the profile of second-hand smoking exposure potentially heterogeneous in LAC region, deserving a more contemporary assessment.

Therefore, the present study aimed to describe the prevalence of second-hand smoke exposure and daily second-hand smoking in the LAC region, overall and by sex. In addition, subregion analyses were also performed to better understand the epidemiology of second-hand smoking in the LAC.

## Methods

### Study design

We utilised information from the Global School-based Student Health (GSHS), a group of surveys built to assess behavioural factors among adolescents between 13 and 17 years. Data from 2010 to 2018, from countries of the Latin American and the Caribbean region, were pooled for analysis.

### Survey characteristics

The GSHS was developed by the World Health Organization (WHO), with help of the Centers for Disease Control and Prevention (CDC) of the United States, and other partners, and data is freely accessible.^18^

The GSHS is conducted independently in each participating country and includes a core questionnaire to assess 10 key areas (alcohol use, dietary behaviours, drug use, hygiene, mental health, physical activity, protective factors, sexual behaviours, tobacco use, and violence and unintentional injury), core-expanded questions, and country-specific questions.^19^

For the selection of participants in each country, the survey utilises a bietapic sampling technique. Thus, in the first phase, the selection of schools is proportional to the sample size; whilst in the second phase, classrooms within each of the selected schools are randomly chosen. All the students of that selected classroom are eligible to participate.^18^

### Definition of variables

Two variables were the outcome of interest based on a specific question from the tobacco use core module: “During the past 7 days, on how many days have people smoked in your presence?”. Response options were 0 days, 1 or 2 days, 3 or 4 days, 5 or 6 days, and all 7 days. For analysis purposes, two different variables were created to assess exposure to second-hand smoke: the first one was based on any exposure, and split subjects into two groups, one with no exposure (i.e., 0 days) against those with any exposure to second-hand smoke (i.e., ≥1 day). The second variable was based on daily exposure to second-hand smoke, i.e., those <7 days vs those with all the 7 days of exposure. This approach was based on previous literature regarding this topic.^4^^,^^6^^,^^20^

Additionally, another question of the tobacco use module was utilised: “How old were you when first tried a cigarette?” and those who responded “I have never smoked cigarettes” were considered as those without history of smoking (i.e., never smokers) for sensitivity analyses purposes. Other variables included in the analysis for description purposes were: sex (female vs male), age (in years), country, and survey year.

Finally, the participating countries were grouped into four subregions within LAC using an adapted version of the NCD Risk Factor Collaboration approach^21^^,^^22^: Andean Latin America, Caribbean, Central Latin America, and Southern Latin America (Supplementary Table S1).

### Statistical analysis

STATA 16 for Windows (StataCorp, College Station, TX, US) was used for statistical analysis. All the analyses were done considering the multistage design of each survey by using the denormalized individual GSHS survey weight, and taking into account the sampling design and non-response rates. Analyses by sex were conducted using the appropriate subpopulation option in STATA.^23^

Age-standardised prevalence of the outcomes of interest was estimated using the WHO population as standard. Such estimations were carried out overall, by sex, by country, and by subregion. Additionally, a sensitivity analysis was pursued by including only those with no history of previous smoking.

Estimates were compared using the Chi-squared test with the second-order correction of Rao and Scott for categorical variables.^24^ A p-value <0.05 was considered as statistically significant.

### Ethics

Data of the survey is freely available without personal identifiers. As a result ethical review was not considered mandatory.

### Role of the funding source

The funder of the study had no role in study design, data collection, data analysis, data interpretation, or writing of the report.

## Results

### Overall description of the study population

Surveys were carried out between 2010 (Guyana and Peru) and 2018 (Argentina, Panama, St. Lucia, and St. Vincent & Grenadines). Sample sizes varied from 813 in Anguilla (2016) to 56,981 in Argentina (2018), adding up to a total of 95,805 records in 18 LAC countries (See Table 1).

**Table 1:** Data available for statistical analyses (n = 95,805).

Country	Study year	Sample size	Missing values (%)	Age, mean (SD)	Female (%)
Anguilla	2016	813	7.5%	14.6 (1.2)	52.5%
Argentina	2018	56981	4.4%	14.6 (1.1)	52.3%
Bahamas	2013	1357	7.1%	13.5 (1.0)	52.4%
Barbados	2011	1629	5.6%	14.3 (0.9)	50.8%
Bolivia	2012	3696	11.7%	14.5 (1.1)	49.5%
Chile	2013	2049	10.8%	14.6 (1.4)	51.1%
Curacao	2015	2765	23.7%	14.7 (1.3)	50.7%
Dominican Republic	2016	1481	15.4%	15.0 (1.1)	50.5%
Guyana	2010	2392	9.2%	14.4 (1.0)	52.1%
Honduras	2012	1779	8.5%	13.9 (1.3)	53.4%
Jamaica	2017	1667	10.6%	15.0 (1.1)	52.7%
Panama	2018	2948	13.3%	14.9 (1.1)	52.6%
Peru	2010	2882	6.7%	14.4 (1.0)	50.2%
St Lucia	2018	1970	11.9%	14.3 (1.4)	52.8%
St Vincent & Grenadines	2018	1877	14.4%	15.0 (1.0)	51.9%
Suriname	2016	2126	12.8%	14.3 (1.3)	51.2%
Trinidad & Tobago	2017	3869	13.1%	14.2 (1.4)	52.3%
Uruguay	2012	3524	4.2%	14.4 (1.0)	54.7%

Missing values included only key variables for analysis.

Missing values in key variables for analyses were present in 7.2% of the records, ranging from 4.2% in Uruguay (2012) to 23.7% in Curacao (2015). Pooled mean age was 14.6 (SD: 1.2) years, varying from 13.5 in Bahamas to 15.0 years in Dominican Republic, Jamaica, and St. Vincent and Grenadines. The overall proportion of girls was 51.5%, varying from 49.5% in Bolivia to 54.7% in Uruguay.

### Second-hand smoke exposure

**Fig. 1:**
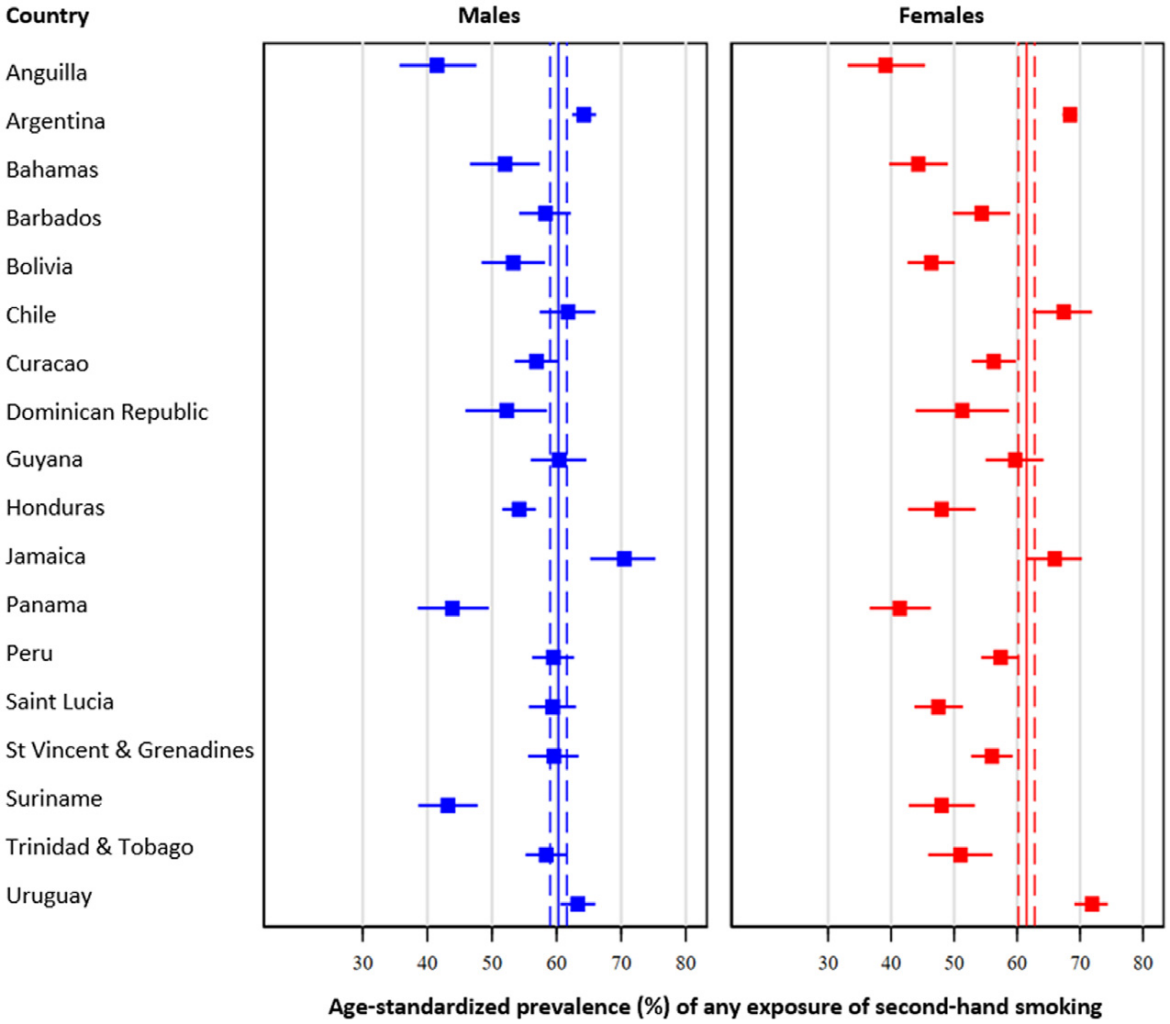
**Age-standardised prevalence of any exposure of second-hand smoking: Results by country and sex.** Pooled age-standardised prevalence is shown as continuous line (point estimate) and dashed lines (95% confidence intervals).

Pooled age-standardised prevalence of second-hand smoke exposure was 60.9% (95% CI: 59.9%–62.0%); however, prevalence estimates varied from 40.2% (95% CI: 35.2%–45.5%) in Anguilla to 68.2% (95% CI: 64.5%–71.6%) in Jamaica (See details in Fig. 1 and Supplementary Table S2). When analyses were done by subregion, the highest prevalence of second-hand smoke exposure was in the Southern Latin America subregion (65.9%), followed by the Caribbean (56.0%), Andean Latin America (55.7%), and finally Central Latin America (48.4%, p < 0.001).

Pooled estimates were similar between males (60.3%; 95% CI: 59.0%–61.6%) compared to females (61.5%; 95% CI: 60.2%–62.8%, p = 0.12). Second-hand smoke exposure was greater among males compared to females in Andean Latin America (57.5% vs 53.9%, p = 0.02) and Central Latin America (51.0% vs 46.0%, p = 0.01); whereas the situation was inverse in Southern Latin America (63.4% vs 68.2%, p < 0.001). There was no difference in second-hand smoke exposure in the Caribbean subregion (57.2% in males vs 54.9% in females, p = 0.28).

When the analysis was conducted among never smokers, the prevalence of second-hand smoke exposure was 51.5% (95% CI: 50.4%–52.6%). The lowest prevalence was in Anguilla (36.3%; 95% CI: 31.5%–41.4%) and Panama (36.3%; 95% CI: 31.6%–41.3%), whereas the highest prevalence was in Jamaica (62.2%; 95% CI: 57.7%–66.4%). By sex, second-hand smoke was slightly lower among males (50.6%; 95% CI: 49.1%–52.1%) than females (52.3%; 95% CI: 50.8%–53.8%), but this difference was not significant (p = 0.09). By subregion, the prevalence of second-hand smoke exposure was 54.9% in Southern Latin America, followed by the Caribbean (50.3%), Andean Latin America (48.4%) and Central Latin America (42.5%). Detailed information by country and sex can be seen in Supplementary Figure S1 and Supplementary Table S3.

### Daily second-hand smoke exposure

Pooled age-standardised prevalence of daily second-hand smoke exposure was 15.1% (95% CI: 14.2%–16.1%); however, prevalence estimates varied from 4.8% (95% CI: 3.5%–6.4%) in Peru to 28.7% (95% CI: 24.1%–33.8%) in Jamaica. When analyses were done by subregion, the highest prevalence of daily second-hand smoke exposure was in Southern Latin America (19.7%), followed by the Caribbean (17.2%), Central Latin America (11.1%), and Andean Latin America (4.9%, p < 0.001).

**Fig. 2:**
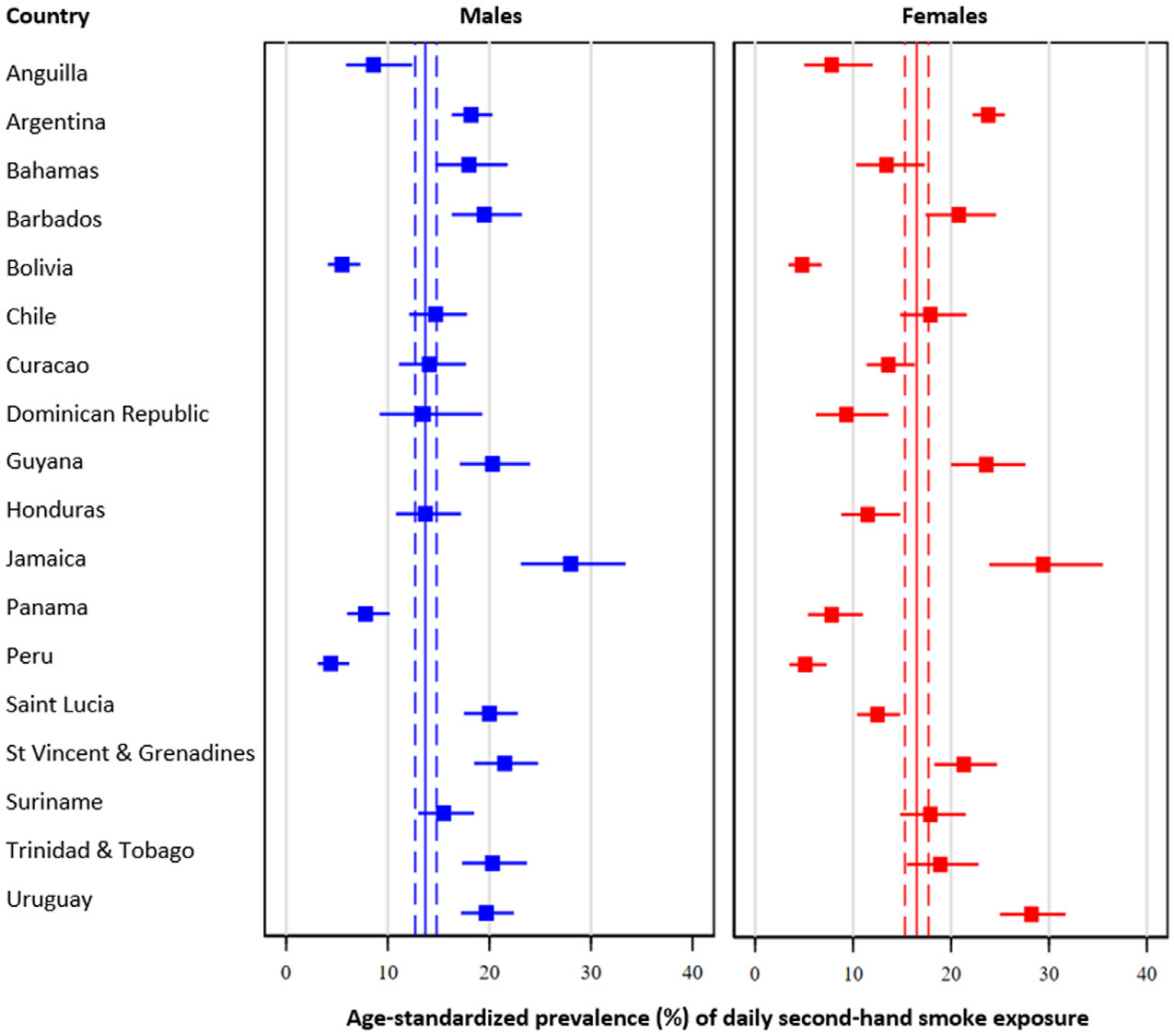
**Age-standardised prevalence of daily second-hand smoke exposure: Results by country and sex.** Pooled age-standardised prevalence is shown as continuous line (point estimate) and dashed lines (95% confidence intervals).

Pooled estimates were lower among males (13.7%; 95% CI: 12.7%–14.8%) compared to females (16.5%; 95% CI: 15.3%–17.7%, p < 0.001). See details in Fig. 2 and Supplementary Table S4. Daily second-hand smoke exposure was lower among males compared to females only in the Southern Latin America (17.1% vs 22.1%, p < 0.001), whereas there was no difference in the other subregions: 18.1% vs 16.4% in the Caribbean (p = 0.21), 11.9% vs 10.4% (p = 0.23) in Central Latin America, and 4.7% vs 5.0% (p = 0.71) in Andean Latin America, when males were compared to females, respectively.

When the analysis was conducted among never smokers, the prevalence of daily second-hand smoke exposure was 9.8% (95% CI: 9.1%–10.7%). The lowest prevalence was in Peru (3.4%; 95% CI: 2.1%–5.3%), whereas the highest prevalence was in Jamaica (21.1%; 95% CI: 16.8%–21.2%). By sex, daily second-hand smoke was lower among males (9.1%; 95% CI: 8.1%–10.2%) than females (10.5%; 95% CI: 9.6%–11.5%, p = 0.02). By subregion, the prevalence of daily second-hand smoke exposure was 12.7% in Southern Latin America, followed by the Caribbean (12.4%), Central Latin America (7.9%), and Andean Latin America (3.6%). Information by country and sex can be seen in Supplementary Figure S2 and Supplementary Table S5.

## Discussion

### Main findings

Our results confirm that about 60% of adolescents between 13 and 17 years reported any second-hand smoke exposure, whereas 15% were daily exposed to second-hand smoking. These rates dropped, but are still high (52% and 10% for any and daily second-hand smoking, respectively), when only never smokers were analysed. Although adolescents of both sexes were similarly exposed, females had greater daily second-hand smoking rates compared to males. Finally, second-hand smoke exposure was greater among adolescents from the Southern Latin America subregion.

### Comparison with previous studies

Our estimates of any second-hand smoking prevalence using the GSHS are similar to those obtained in a global analysis using the Global Youth Tobacco Survey (GYTS) in the same period (2010–2018), but lower to that obtained for the Americas region.^4^ Similarly, estimates by sex were almost the same, and no difference between sexes was found.^4^^,^^5^ Nevertheless, the overall prevalence of daily second-hand smoking was half of the estimate reported in the global analysis using the GYTS, several percentual points below the estimate in the Americas region,^4^ and greater among females than males. Although the question utilised for estimating second-hand smoking prevalence was similar, differences may be attributed to the fact that our prevalence estimates were age-standardised whereas previous results were not. In addition, countries involved in the GSHS are not the same from those included in the GYTS, highlighting the existing heterogeneity in the region.

Our study expands on previous findings by estimating the prevalence of second-hand smoke exposure among never smokers. A previous global study, using information of the GYTS from 1999 to 2008, reported a prevalence of second-hand smoking of 23% both inside and outside home,^6^ a value higher compared to our estimate. According to this latter study, the estimate in the LAC region was 22%, which was also higher compared to our findings. So, a potential reduction in rates of daily second-hand smoking may have been occurred over time, but the differences may be also attributed to the surveys’ characteristics as previously mentioned. A different study,^20^ analysing information from 31 countries, reported that approximately half of children (<11 years) were exposed to second-hand smoking outside the household, and this estimate was 48% for the North and South America region. Moreover, this study found that second-hand smoking was almost the rule in household with smokers, which usually tend to smoke around their children with little restraint.

### Public health relevance

More than half of never-smoker adolescents were exposed to second-hand smoking during the last seven days, and one in ten were daily exposed, with higher exposure among females. These findings are relevant as second-hand smoke exposure has been linked to an increased probability of being a smoker,^7^ increased susceptibility to smoking,^25^ increased probability of smoking initiation,^26^ greater nicotine dependence symptoms,^8^ reduced success in smoking cessation,^10^ and deleterious health outcomes.^27^

Whereas tobacco control policies have improved in many LAC countries, especially those related to protection against tobacco smoke at home and selling cigarettes to adolescents, only small reductions were seen in second-hand smoking outside home.^3^ Thus, airborne nicotine has been detected in more than 90% of indoor public environments across different locations surveyed in cities in the LAC region.^17^^,^^28^ In addition to that, exposure to media and advertising remained largely unchanged.^3^

As the spread of tobacco is facilitated by a variety of complex factors with cross-border effects, including trade liberalisation and globalisation,^29^ the heterogeneity observed in second-hand smoking rates across countries suggest that efforts to control tobacco exposure may be advancing at different pace in the region. According to our findings, a more continuous and consistent surveillance of second-hand smoking in LAC countries is needed, as well as strengthening efforts to better control tobacco use.

### Strengths and limitations

This study has several strengths. We used the most updated data of the GSHS (i.e., from 2010 to 2018) for 18 countries of the LAC region. Moreover, the same questions regarding second-hand smoking and smoke exposure were utilised in all the settings, making feasible the comparison between countries. In addition, we described second-hand smoke exposure among those who reported never smoking, which can be more relevant from the public health perspective.

However, several limitations should be highlighted. First, the GSHS uses a self-report tool to collect behavioural information, which can lead to bias, particularly recall and social desirability bias. Second, only a subgroup of adolescents (i.e., those present at the school during the survey) was evaluated using this strategy, therefore results should be interpreted cautiously, especially as related to generalizability. Third, not all the countries of the LAC region conduct the GSHS. Fourth, we could not assess whether the exposure occurs at home or elsewhere as done in previous works.^4^^,^^6^ Fifth, the surveys were administered in different years (from 2010 to 2018) across countries. On one hand, this may make between-country comparisons difficult, whereas in the other hand, for each country, the prevalence estimates should be interpreted alongside the year when the survey was conducted to better understand the local context. Finally, prevalence estimates were highly heterogeneous. However, we provided pooled estimated by subregions to gain a better epidemiology perspective of this public health problem.

### Conclusions

Although heterogeneous, the prevalence of second-hand smoke exposure is high among adolescents in the LAC region, including among those who never smoked. Adolescents of both sexes were similarly exposed, but females had greater daily second-hand smoking rates compared to males. There is a need to strengthen tobacco control policies in the region.

## Contributors

AB-O and RMC-L conceived the idea of the manuscript. AB-O conducted the statistical analysis with support of RMC-L. AB-O drafted the first version of the manuscript with critical input of RMC-L. Both authors approved the final version submitted for publication. AB-O and RMC-L had full access to the data and conducted the statistical analyses, and they are the guarantors of the study and vouch for the accuracy of the results. AB-O had final responsibility for the decision to submit for publication.

## Data sharing statement

Data of the GSHS is freely available at the WHO NCD Microdata Repository website: https://extranet.who.int/ncdsmicrodata/index.php/catalog/GSHS/about.

## Declaration of interests

The authors declare that no conflicts of interest exist.

## Appendix A Supplementary data

Supplementary Figures and Tables

Translated Abstract (Spanish)
