# Supplementary material for: Second-hand smoke exposure in adolescents in Latin America and the Caribbean: a pooled analysis
Source: Lancet Reg Health Am. 2023 Mar 20;20:100478. doi: 10.1016/j.lana.2023.100478 (PMC10033735; doi:10.1016/j.lana.2023.100478)
Supplement: Supplementary Figures and Tables [file mmc1.docx]

**SUPPLEMENTARY FILE**

**Supplementary Figure 1. Age-standardized prevalence of any exposure to second-hand smoking among never smokers: Results by country and sex.** Pooled age-standardized prevalence is shown as continuous line (point estimate) and dashed lines (95% confidence intervals).

**Supplementary Figure 2. Age-standardized prevalence of daily second-hand smoke exposure among never smokers: Results by country and sex.** Pooled age-standardized prevalence is shown as continuous line (point estimate) and dashed lines (95% confidence intervals).

**Supplementary Table 1. Subregions in the Latin American and the Caribbean**

**Supplementary Table 2. Age-standardized prevalence of any exposure to second-hand smoking: by sex and country.**

**Supplementary Table 3. Age-standardized prevalence of any exposure to second-hand smoking among never smokers: by sex and country**

**Supplementary Table 4. Age-standardized prevalence of daily second-hand smoke exposure: by sex and country**

**Supplementary Table 5. Age-standardized prevalence of daily second-hand smoke exposure among never smokers: by sex and country**

**Supplementary Figure 1.** **Age-standardized prevalence of any exposure to second-hand smoking among never smokers: Results by country and sex**

| **Country** | **Males** | **Females** |
| --- | --- | --- |
| Anguilla | 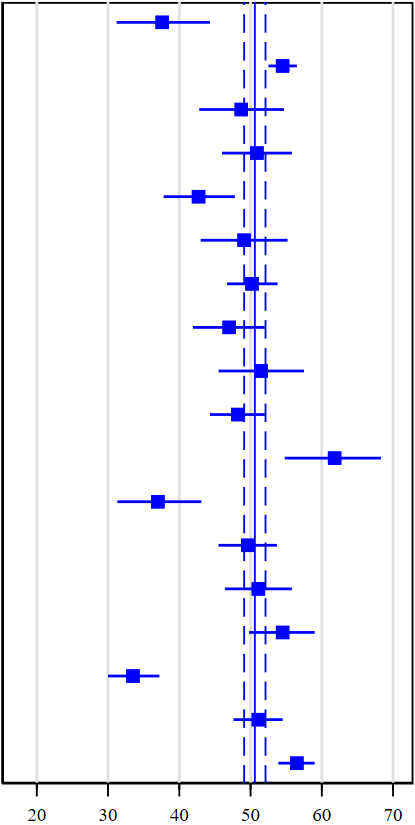 | 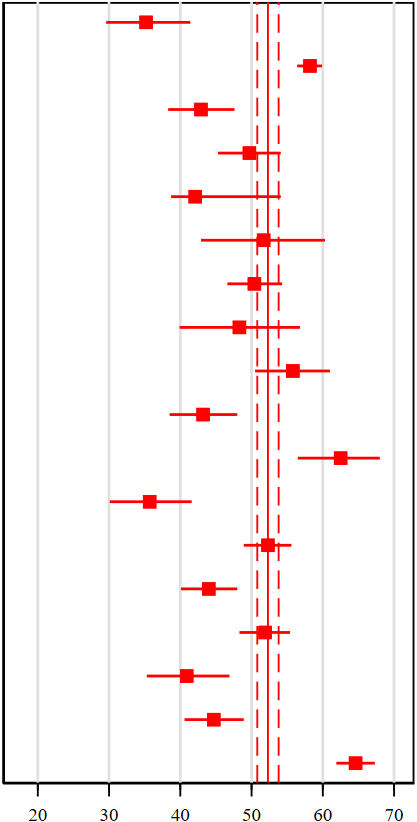 |
| Argentina |  |  |
| Bahamas |  |  |
| Barbados |  |  |
| Bolivia |  |  |
| Chile |  |  |
| Curacao |  |  |
| Dominican Republic |  |  |
| Guyana |  |  |
| Honduras |  |  |
| Jamaica |  |  |
| Panama |  |  |
| Peru |  |  |
| Saint Lucia |  |  |
| St Vincent & Grenadines |  |  |
| Suriname |  |  |
| Trinidad & Tobago |  |  |
| Uruguay |  |  |
|  |  |  |
|  | **Age-standardized prevalence (%) of any exposure of second-hand smoking** | |

Pooled age-standardized prevalence is shown as continuous line (point estimate) and dashed lines (95% confidence intervals).

**Supplementary Figure 2.** **Age-standardized prevalence of daily second-hand smoke exposure among never smokers: Results by country and sex**

| **Country** | **Males** | **Females** |
| --- | --- | --- |
| Anguilla | 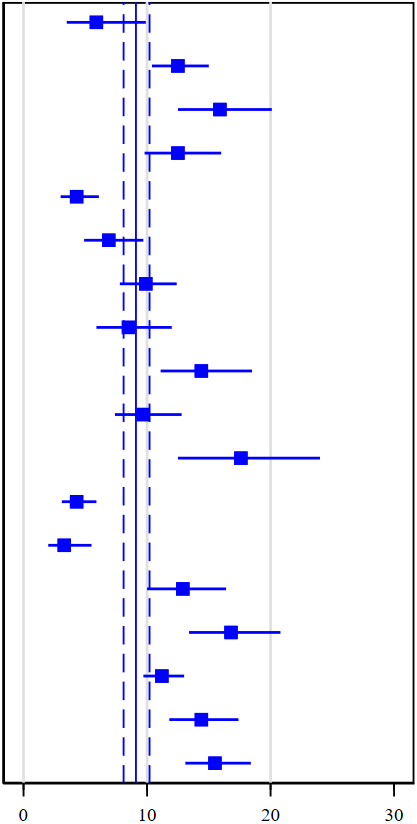 | 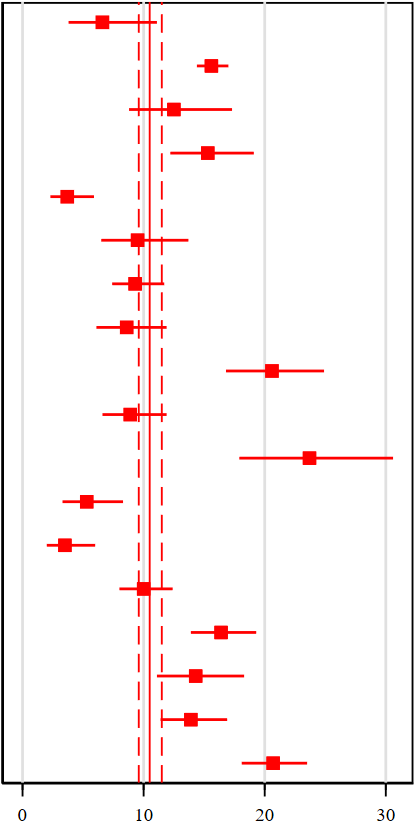 |
| Argentina |  |  |
| Bahamas |  |  |
| Barbados |  |  |
| Bolivia |  |  |
| Chile |  |  |
| Curacao |  |  |
| Dominican Republic |  |  |
| Guyana |  |  |
| Honduras |  |  |
| Jamaica |  |  |
| Panama |  |  |
| Peru |  |  |
| Saint Lucia |  |  |
| St Vincent & Grenadines |  |  |
| Suriname |  |  |
| Trinidad & Tobago |  |  |
| Uruguay |  |  |
|  |  |  |
|  | **Age-standardized prevalence (%) of continuous exposure of second-hand smoking** | |

Pooled age-standardized prevalence is shown as continuous line (point estimate) and dashed lines (95% confidence intervals).

**Supplementary Table 1.** **Subregions in the Latin American and the Caribbean**

| **Andean Latin America (2)** | Bolivia, Peru |
| --- | --- |
| **Caribbean (11)** | Anguilla, Bahamas, Barbados, Curacao, Dominican Republic, Guyana, Jamaica, St Lucia, St Vincent and the Grenadines, Suriname, Trinidad and Tobago |
| **Central Latin America (2)** | Honduras, Panama |
| **Southern Latin America (3)** | Argentina, Chile, Uruguay |

**Supplementary Table 2.** **Age-standardized prevalence of any exposure to second-hand smoking: by sex and country**

| **Country** | **Second-hand smoking (any exposure)** | | |
| --- | --- | --- | --- |
|  | **Total** | **Male** | **Female** |
| Anguilla | 40.2% (35.2% - 45.5%) | 41.5% (35.7% - 47.6%) | 39.1% (33.1% - 45.4%) |
| Argentina | 66.4% (65.1% - 67.6%) | 64.2% (62.4% - 66.1%) | 68.4% (67.2% - 69.5%) |
| Bahamas | 48.0% (43.2% - 52.8%) | 52.0% (46.6% - 57.4%) | 44.3% (39.7% - 49.0%) |
| Barbados | 56.3% (52.9% - 59.7%) | 58.3% (54.2% - 62.2%) | 54.4% (49.8% - 58.9%) |
| Bolivia | 49.9% (46.3% - 53.5%) | 53.3% (48.4% - 58.2%) | 46.4% (42.6% - 50.1%) |
| Chile | 64.7% (61.1% - 68.0%) | 61.8% (57.4% - 66.0%) | 67.4% (62.5% - 71.9%) |
| Curacao | 56.6% (54.1% - 59.1%) | 56.9% (53.5% - 60.2%) | 56.3% (52.8% - 59.8%) |
| Dominican Republic | 51.8% (46.2% - 57.3%) | 52.3% (45.9% - 58.5%) | 51.3% (43.9% - 58.7%) |
| Guyana | 60.0% (56.2% - 63.8%) | 60.4% (56.0% - 64.6%) | 59.7% (55.0% - 64.2%) |
| Honduras | 50.9% (47.4% - 54.4%) | 54.2% (51.6% - 56.8%) | 48.0% (42.7% - 53.4%) |
| Jamaica | 68.2% (64.5% - 71.6%) | 70.5% (65.2% - 75.3%) | 66.0% (61.4% - 70.3%) |
| Panama | 42.6% (38.2% - 47.1%) | 43.9% (38.5% - 49.5%) | 41.4% (36.6% - 46.3%) |
| Peru | 58.4% (56.0% - 60.8%) | 59.5% (56.2% - 62.7%) | 57.4% (54.3% - 60.3%) |
| St Lucia | 53.1% (50.1% - 56.1%) | 59.4% (55.7% - 63.0%) | 47.5% (43.7% - 51.4%) |
| St Vincent & Grenadines | 57.7% (55.1% - 60.4%) | 59.6% (55.6% - 63.4%) | 56.0% (52.7% - 59.3%) |
| Suriname | 45.7% (41.8% - 49.6%) | 43.2% (38.6% - 47.8%) | 48.0% (42.8% - 53.3%) |
| Trinidad & Tobago | 54.5% (51.2% - 57.8%) | 58.4% (55.2% - 61.5%) | 51.0% (45.9% - 56.1%) |
| Uruguay | 68.0% (66.0% - 69.9%) | 63.3% (60.6% - 66.0%) | 71.9% (69.1% - 74.4%) |

**Supplementary Table 3.** **Age-standardized prevalence of any exposure to second-hand smoking among never smokers: by sex and country**

| **Country** | **Second-hand smoking (any exposure)**  **among those without history of smoking** | | |
| --- | --- | --- | --- |
|  | **Total** | **Male** | **Female** |
| Anguilla | 36.3% (31.5% - 41.4%) | 37.6% (31.2% - 44.3%) | 35.2% (29.6% - 41.4%) |
| Argentina | 56.4% (55.1% - 57.7%) | 54.5% (52.5% - 56.5%) | 58.2% (56.4% - 59.9%) |
| Bahamas | 45.4% (40.6% - 50.4%) | 48.7% (42.8% - 54.7%) | 42.9% (38.3% - 47.6%) |
| Barbados | 50.3% (46.7% - 53.8%) | 50.9% (46.0% - 55.8%) | 49.7% (45.3% - 54.1%) |
| Bolivia | 42.4% (39.2% - 45.6%) | 42.7% (37.8% - 47.8%) | 42.1% (38.7% - 45.7%) |
| Chile | 50.3% (45.5% - 55.1%) | 49.1% (43.0% - 55.2%) | 51.7% (42.9% - 60.3%) |
| Curacao | 50.3% (47.6% - 53.0%) | 50.2% (46.7% - 53.8%) | 50.4% (46.6% - 54.3%) |
| Dominican Republic | 47.7% (42.1% - 53.4%) | 47.0% (41.9% - 52.0%) | 48.3% (39.9% - 56.8%) |
| Guyana | 54.1% (50.0% - 58.1%) | 51.5% (45.5% - 57.5%) | 55.8% (50.5% - 61.0%) |
| Honduras | 45.3% (41.7% - 49.1%) | 48.2% (44.3% - 52.0%) | 43.2% (38.5% - 48.0%) |
| Jamaica | 62.2% (57.7% - 66.4%) | 61.8% (54.8% - 68.3%) | 62.5% (56.5% - 68.0%) |
| Panama | 36.3% (31.6% - 41.3%) | 37.0% (31.3% - 43.1%) | 35.7% (30.1% - 41.6%) |
| Peru | 51.2% (48.3% - 54.0%) | 49.6% (45.5% - 53.7%) | 52.3% (48.9% - 55.6%) |
| St Lucia | 47.0% (43.7% - 50.4%) | 51.1% (46.4% - 55.8%) | 44.0% (40.1% - 48.0%) |
| St Vincent & Grenadines | 53.0% (50.1% - 55.9%) | 54.5% (49.8% - 59.0%) | 51.9% (48.3% - 55.4%) |
| Suriname | 37.5% (34.0% - 41.1%) | 33.5% (30.0% - 37.2%) | 40.9% (35.3% - 46.9%) |
| Trinidad & Tobago | 47.5% (44.6% - 50.4%) | 51.1% (47.6% - 54.5%) | 44.7% (40.6% - 48.9%) |
| Uruguay | 60.8% (58.7% - 62.7%) | 56.5% (53.9% - 59.0%) | 64.6% (61.9% - 67.3%) |

**Supplementary Table 4.** **Age-standardized prevalence of daily second-hand smoke exposure: by sex and country**

| **Country** | **Second-hand smoking (continuous exposure)** | | |
| --- | --- | --- | --- |
|  | **Total** | **Male** | **Female** |
| Anguilla | 8.2% (6.1% - 10.9%) | 8.6% (5.9% - 12.4%) | 7.8% (5.0% - 12.0%) |
| Argentina | 21.1% (19.6% - 22.7%) | 18.2% (16.3% - 20.3%) | 23.8% (22.2% - 25.5%) |
| Bahamas | 15.6% (12.8% - 18.9%) | 18.0% (14.7% - 21.8%) | 13.4% (10.3% - 17.3%) |
| Barbados | 20.2% (17.9% - 22.6%) | 19.5% (16.3% - 23.2%) | 20.8% (17.4% - 24.6%) |
| Bolivia | 5.2% (4.0% - 6.7%) | 5.5% (4.1% - 7.3%) | 4.8% (3.4% - 6.8%) |
| Chile | 16.4% (14.0% - 19.0%) | 14.7% (12.1% - 17.8%) | 17.9% (14.8% - 21.6%) |
| Curacao | 13.9% (11.7% - 16.3%) | 14.1% (11.1% - 17.7%) | 13.6% (11.4% - 16.3%) |
| Dominican Republic | 11.3% (8.3% - 15.3%) | 13.5% (9.2% - 19.3%) | 9.3% (6.2% - 13.6%) |
| Guyana | 22.0% (19.3% - 25.1%) | 20.3% (17.1% - 24.0%) | 23.6% (20.0% - 27.6%) |
| Honduras | 12.5% (10.2% - 15.2%) | 13.7% (10.8% - 17.2%) | 11.5% (8.8% - 14.8%) |
| Jamaica | 28.7% (24.1% - 33.8%) | 28.0% (23.1% - 33.4%) | 29.4% (23.9% - 35.5%) |
| Panama | 7.8% (5.9% - 10.3%) | 7.8% (6.0% - 10.2%) | 7.8% (5.4% - 11.0%) |
| Peru | 4.8% (3.5% - 6.4%) | 4.4% (3.1% - 6.2%) | 5.1% (3.5% - 7.3%) |
| St Lucia | 16.0% (14.2% - 18.0%) | 20.0% (17.5% - 22.8%) | 12.5% (10.4% - 14.8%) |
| St Vincent & Grenadines | 21.4% (19.3% - 23.7%) | 21.5% (18.5% - 24.8%) | 21.3% (18.3% - 24.7%) |
| Suriname | 16.8% (14.7% - 19.1%) | 15.5% (13.0% - 18.5%) | 17.9% (14.8% - 21.5%) |
| Trinidad & Tobago | 19.6% (17.1% - 22.3%) | 20.3% (17.3% - 23.7%) | 18.9% (15.5% - 22.8%) |
| Uruguay | 24.3% (21.9% - 27.0%) | 19.7% (17.2% - 22.4%) | 28.2% (25.0% - 31.7%) |

**Supplementary Table 5.** **Age-standardized prevalence of daily second-hand smoke exposure among never smokers: by sex and country**

| **Country** | **Second-hand smoking (continuous exposure)**  **among those without history of smoking** | | |
| --- | --- | --- | --- |
|  | **Total** | **Male** | **Female** |
| Anguilla | 6.3% (4.2% - 9.2%) | 5.9% (3.5% - 9.9%) | 6.6% (3.8% - 11.1%) |
| Argentina | 14.1% (12.7% - 15.7%) | 12.5% (10.4% - 15.0%) | 15.6% (14.4% - 17.0%) |
| Bahamas | 14.0% (11.0% - 17.6%) | 15.9% (12.5% - 20.1%) | 12.5% (8.8% - 17.3%) |
| Barbados | 14.0% (11.8% - 16.6%) | 12.5% (9.8% - 16.0%) | 15.3% (12.2% - 19.1%) |
| Bolivia | 4.0% (2.8% - 5.5%) | 4.3% (3.0% - 6.1%) | 3.7% (2.3% - 5.9%) |
| Chile | 8.2% (6.5% - 10.2%) | 6.9% (4.9% - 9.7%) | 9.5% (6.5% - 13.7%) |
| Curacao | 9.6% (8.0% - 11.4%) | 9.9% (7.8% - 12.4%) | 9.3% (7.4% - 11.7%) |
| Dominican Republic | 8.5% (6.6% - 10.9%) | 8.5% (5.9% - 12.0%) | 8.6% (6.1% - 11.9%) |
| Guyana | 18.0% (15.3% - 21.2%) | 14.4% (11.1% - 18.5%) | 20.6% (16.8% - 24.9%) |
| Honduras | 9.3% (7.5% - 11.4%) | 9.7% (7.4% - 12.8%) | 8.9% (6.6% - 11.9%) |
| Jamaica | 21.1% (16.8% - 26.1%) | 17.6% (12.5% - 24.0%) | 23.7% (17.9% - 30.6%) |
| Panama | 4.8% (3.4% - 6.7%) | 4.3% (3.1% - 5.9%) | 5.3% (3.3% - 8.3%) |
| Peru | 3.4% (2.1% - 5.3%) | 3.3% (2.0% - 5.5%) | 3.5% (2.0% - 6.0%) |
| St Lucia | 11.2% (9.4% - 13.4%) | 12.9% (10.0% - 16.4%) | 10.0% (8.0% - 12.4%) |
| St Vincent & Grenadines | 16.6% (14.6% - 18.8%) | 16.8% (13.4% - 20.8%) | 16.4% (13.9% - 19.3%) |
| Suriname | 12.9% (10.9% - 15.3%) | 11.2% (9.7% - 13.0%) | 14.3% (11.1% - 18.3%) |
| Trinidad & Tobago | 14.1% (12.2% - 16.3%) | 14.4% (11.8% - 17.4%) | 13.9% (11.4% - 16.9%) |
| Uruguay | 18.2% (16.3% - 20.3%) | 15.5% (13.1% - 18.4%) | 20.7% (18.1% - 23.5%) |
