# Supplementary material for: Second-hand smoke exposure in adolescents in Latin America and the Caribbean: a pooled analysis
Source: Lancet Reg Health Am. 2023 Mar 20;20:100478. doi: 10.1016/j.lana.2023.100478 (PMC10033735; doi:10.1016/j.lana.2023.100478)
Supplement: Translated Abstract (Spanish) [file mmc2.docx]

*Editor note: This translation in Spanish was submitted by the authors and we reproduce it as supplied. It has not been peer reviewed. Our editorial processes have only been applied to the original abstract in English, which should serve as reference for this manuscript*

**RESUMEN**

**Antecedentes:** El tabaquismo pasivo es prevalente en los adolescentes, a pesar de que es un factor de riesgo prevenible asociado a desenlaces desfavorables. La distribución de este factor de riesgo varía según los determinantes subyacentes, y los funcionarios de salud pública necesitan evidencia contemporánea para actualizar las políticas. Usando los datos más recientes disponibles de adolescentes en América Latina y el Caribe (ALC), describimos la prevalencia del tabaquismo pasivo.

**Métodos:** Se realizó un análisis combinado de las encuestas GSHS realizadas entre 2010 y 2018. Se analizaron dos indicadores basados en la información de los 7 días previos a la encuesta: a) cualquier exposición al tabaquismo pasivo (0 vs. ≥1 días de exposición); y b) exposición diaria (<7 vs. a 7 días). Las estimaciones de prevalencia se realizaron teniendo en cuenta el complejo diseño de las encuestas y se informaron en general, por país, por sexo y por subregión.

**Hallazgos:** Las encuestas GSHS se administraron en 18 países, lo que arrojó un total de 95.805 sujetos. La prevalencia general estandarizada por edad del tabaquismo pasivo fue del 60,9 % (IC del 95 %: 59,9 %-62,0 %) sin diferencias sustanciales entre niños y niñas. La prevalencia estandarizada por edad de cualquier tabaquismo pasivo varió del 40,2 % en Anguila al 68,2 % en Jamaica, y la prevalencia más alta se registró en la subregión del Sur de América Latina (65,9 %). La prevalencia general estandarizada por edad del tabaquismo pasivo diario fue del 15,1 % (IC del 95 %: 14,2 %-16,1 %) y fue mayor en las niñas que en los niños (16,5 % frente a 13,7 %; p<0,001). La prevalencia estandarizada por edad del tabaquismo pasivo diario osciló entre 4,8 % en Perú y 28,7 % en Jamaica, y la prevalencia estandarizada por edad más alta se registró en el sur de América Latina (19,7 %).

**Interpretación:** La prevalencia de cualquier tipo de tabaquismo pasivo es alta entre los adolescentes de ALC, aunque las estimaciones cambiaron sustancialmente según el país. Si bien se han implementado políticas e intervenciones para reducir/dejar de fumar, también se debe prestar atención evitar la exposición al humo de segunda mano.
